# Ground-State Properties and Phase Separation of Binary Mixtures in Mesoscopic Ring Lattices

**DOI:** 10.3390/e23070821

**Published:** 2021-06-28

**Authors:** Vittorio Penna, Alessandra Contestabile, Andrea Richaud

**Affiliations:** 1 Department of Applied Science and Technology and u.d.r. CNISM, Politecnico di Torino, 10129 Torino, Italy; alessandra.contestabile@polito.it; 2 Scuola Internazionale Superiore di Studi Avanzati (SISSA), Via Bonomea 265, 34136 Trieste, Italy; arichaud@sissa.it

**Keywords:** boson binary mixtures, phase separation mechanism, lattice size

## Abstract

We investigated the spatial phase separation of the two components forming a bosonic mixture distributed in a four-well lattice with a ring geometry. We studied the ground state of this system, described by means of a binary Bose–Hubbard Hamiltonian, by implementing a well-known coherent-state picture which allowed us to find the semi-classical equations determining the distribution of boson components in the ring lattice. Their fully analytic solutions, in the limit of large boson numbers, provide the boson populations at each well as a function of the interspecies interaction and of other significant model parameters, while allowing to reconstruct the non-trivial architecture of the ground-state four-well phase diagram. The comparison with the *L*-well (L=2,3) phase diagrams highlights how increasing the number of wells considerably modifies the phase diagram structure and the transition mechanism from the full-mixing to the full-demixing phase controlled by the interspecies interaction. Despite the fact that the phase diagrams for L=2,3,4 share various general properties, we show that, unlike attractive binary mixtures, repulsive mixtures do not feature a transition mechanism which can be extended to an arbitrary lattice of size *L*.

## 1. Introduction

The demixing effect in bosonic binary mixtures trapped in optical lattices has attracted a fair amount of attention in the last two decades due to both the rich phenomenology stemming from the spatial separation of mixture components [1,2] and to the considerable complexity that the demixing mechanism features. The latter, basically dominated by the interplay of density–density interspecies and intraspecies repulsions with the particle number of each component, bears memory of the fragmentation of the boson fluid in lattice multiwell structures and of the deeply quantum character of microscopic boson–boson interactions. These features were taken into account by representing boson mixtures and the relevant phenomenology within the well-known second-quantization picture [3] characterizing the Bose–Hubbard (BH) model [4,5]. Experimentally, binary mixtures were realized by either combining different atomic species [6] or by means of components corresponding to different internal states [7,8] of a single atomic species.

Spatial phase separation greatly enriches the phase diagram of mixtures [9,10] characterized by new types of superfluidity and incompressible states [11,12], rules the coexistence of different phases in the presence of trapping potential [13,14], it is involved in the formation of quantum emulsions [15,16] and represents a robust property of mixtures able to survive at non-zero temperature and in spite of the confinement effect [17].

Recently, the miscibility properties of binary mixtures were investigated in small-size optical lattices to obtain a deeper insight into the influence of space fragmentation on the demixing mechanism. The interest for this class of systems was doubly motivated. First, they are within the reach of current experimental techniques. Designed almost fifteen years ago [18], the realization of traps with a ring geometry has been studied in [19,20] to support the development of atomtronics devices, while the technique used for realizing a double well [21,22] indeed supplies a viable scheme to construct linear open arrays of potential wells. Second, the relatively small number of local order parameters necessary to describe the microscopic state of the system often allows one to analytically approach the study of low-energy states and of their properties despite the well-known non-integrable character of BH lattice systems. This, in turn, makes it possible to obtain new information about the miscibility properties and the separation mechanism.

The critical condition for which the interplay of tunneling parameter, boson–boson interactions and boson numbers of the two components links demixing effect and spectral collapse has been analytically investigated in the double-well system [23,24] in the case of both repulsive and attractive components, while the role of inhomogeneity on demixing, due to the trapping effect, has been evidenced by using multiconfigurational time-dependent Hartree method [25]. Purely quantum indicators, such as entanglement and residual entropies [26], and a nontrivial geometric phase [27] have further confirmed the critical behavior distinguishing spatial separation, while the impact of a chaotic dynamical behavior on the robustness of spatial separation has been explored in both lattice structures [28] and in a harmonic trap [29].

More recently, demixing has been investigated for a ring triple well in asymmetric configurations where interspecies and intraspecies repulsions are different, and the imbalance of the two boson populations is non-zero. The higher degree of space fragmentation due to the third well has been clearly shown to imply a more structured phase diagram, and implicitly, a more complex separation mechanism [30,31]. A parallel study on attractive mixtures, whose production has been recently studied in [32] together with the formation of droplet states, has revealed how the transition from mixed fully delocalized components to mixed components confined in a single well (supermixed state) [33] is controlled by the interspecies interaction and the population imbalance, but it is totally independent from the number of potential wells forming the lattice.

This leads to the central problem addressed in this paper. Unlike the attractive mixture, the demixing mechanism of repulsive components seems to feature a strong dependence from boson fragmentation, namely from the number of lattice sites. In particular, any prediction about the structure of the four-well phase diagram can be evinced, apparently, neither from the knowledge of the three-well case nor from some simple or intuitive rule working for an arbitrary site number. This message already emerges from the comparison of the two-well with the three-well phase diagram. Even in the simple case of identical components, the transition from the mixed to demixed state features the occurrence of a third intermediate phase absent in the two-well case. With L=4 (and presumably, with a larger number *L* of wells), the understanding of this mechanism becomes even more problematic.

In this paper, we applied the coherent-state (CS) variational picture to the second-quantized BH model describing the four-well system. This allows us to obtain the ground-state equations and thus to reproduce the mechanism governing the formation of the new phases starting from the weak-interaction regime in which the system was fully mixed and delocalized. We shall compare the phase diagram of the three-well BH model (trimer) with that of the two-well (dimer) and the four-well BH model, with the aim to evidence both the common features shared by the *L*-well systems for L=2,3,4, and the possible diversity they exhibit when demixing, the transition from to full-mixing regime to the complete space separation, is enacted.

Our exploration will be developed by considering the large-population limit, a condition equivalent to assuming vanishingly small effective hopping amplitude in the equations describing the mixture ground state. This approach has been proven to be very effective in investigating both of the three-well phase diagrams [30,31], and the formation of supermixed states in attractive mixtures [33]. The fact that it allows a completely analytic derivation of the phase diagram represents its main feature.

The CS picture of the BH model and the dynamical equations describing the systems in terms of local order parameters is reviewed in Section II. Then, the demixing mechanism of the dimer case, never explored within the current CS-based analytic approach, is discussed in Section III, both to highlight phase separation in its most elementary form, and to exemplify the phase diagram derivation method then applied to the four-well system. Subsection IIA is devoted to revisiting the results concerning the three-well system. Section IV provides a detailed description of the solutions corresponding to five phases occurring in the four-well phase diagram and to the reconstruction of the latter based on the comparison of the ground-state energy of each phase. Finally, Section V is devoted to investigating the onset of critical behaviors and to their characterization by means of the mixing entropy and of the location entropy. The comparison of the properties of the *L*-well phase diagram for L=2,3,4 completes our discussion.

## 2. Coherent-State Picture of BH Model

Binary mixtures loaded in a ring lattice formed by *L* potential wells are well described by the Bose–Hubbard Hamiltonian [3]:

(1) $$\hat{H}={\hat{H}}_{a}+{\hat{H}}_{b}+W\;\sum_{i}\;{\hat{n}}_{ai}{\hat{n}}_{bi}\;,$$

where ${\hat{n}}_{ai}={\hat{a}}_{i}^{+}{\hat{a}}_{i}$ and ${\hat{n}}_{bi}={\hat{b}}_{i}^{+}{\hat{b}}_{i}$ are boson number operators, *W* is the interspecies repulsion strength describing the coupling between the bosons of two components of the mixture, and:

$${\hat{H}}_{c}=\frac{U_{c}}{2}\sum_{i}{\left({\hat{c}}_{i}^{+}\right)}^{2}{\hat{c}}_{i}^{2}-J_{c}\sum_{i}\left({\hat{c}}_{i}^{+}{\hat{c}}_{i+1}+{\hat{c}}_{i+1}^{+}{\hat{c}}_{i}\right)\;,\;\;\;c=a,b$$

is the single-component BH model [5]. Parameters $U_{c}$ and $J_{c}$ represent the boson–boson intraspecies repulsion and the hopping amplitude, respectively, for the component c=a,b. Index *i* ranges within [1,L], where *L* is the site number of the ring lattice, and the boson-mode operators ${\hat{c}}_{i}^{+}$ and ${\hat{c}}_{i}$ satisfy standard commutators $\left[{\hat{c}}_{\ell },{\hat{c}}_{i}^{+}\right]=\delta_{i\ell }$. A distinctive feature of model (1) is that the number operators of each boson component:

$${\hat{N}}_{a}=\sum_{i}{\hat{a}}_{i}^{+}{\hat{a}}_{i},\;{\hat{N}}_{b}=\sum_{i}{\hat{b}}_{i}^{+}{\hat{b}}_{i}$$

are conserved quantities, where $\left[\hat{H},{\hat{N}}_{a}\right]$$=[\hat{H},{\hat{N}}_{b}]=0$, whose eigenvalues $N_{a}$ and $N_{b}$ describe the boson numbers of the two components.

If the system is in the superfluid regime, an assumption guaranteed by the condition that $J_{c}/U_{c}$ (with c=a,b) is sufficiently large, then the dynamical evolution of the bosonic fluid can be represented by means of the coherent-state variational picture [34,35]. In this picture the state of the system is expressed by means of the factorized trial state:

$$|\Phi \rangle =\prod_{i}|a_{i}\rangle \;\prod_{i}|b_{i}\rangle ,$$

and the Glauber coherent states $|a_{i}\rangle$ and $|b_{i}\rangle$ describe the physical state of the two components at the *i*-th well. Within the Glauber-state representation, in fact, the coherent-state labels $a_{i}=\langle a_{i}|{\hat{a}}_{\ell }|a_{i}\rangle$, $b_{i}=\langle b_{i}|{\hat{b}}_{\ell }|b_{i}\rangle$, with $a_{i},b_{i}\in C$, can be interpreted as local order parameters while the expectation value $\langle {\hat{n}}_{ci}\rangle =\langle c_{i}|{\hat{c}}_{i}^{+}{\hat{c}}_{i}|c_{i}\rangle =|c_{i}{|}^{2}$ (c=a,b) represents the average local boson population. The time evolution can be shown to be governed by the discrete nonlinear Schrödinger equations:

(2) $$i\hbar {\dot{a}}_{\ell }=\left(U_{a}|a_{\ell }{|}^{2}+W{\left|b_{\ell }\right|}^{2}\right)a_{\ell }-J_{a}\left(a_{\ell +1}+a_{\ell -1}\right),$$

(3) $$i\hbar {\dot{b}}_{\ell }=\left(U_{b}|b_{\ell }{|}^{2}+W{\left|a_{\ell }\right|}^{2}\right)b_{\ell }-J_{b}\left(b_{\ell +1}+b_{\ell -1}\right).$$

The latter are easily derived from the Hamilton equations:

$${\dot{a}}_{\ell }=\frac{1}{i\hbar }\frac{\partial H}{\partial a_{\ell }^{*}},\;{\dot{b}}_{\ell }=\frac{1}{i\hbar }\frac{\partial H}{\partial b_{\ell }^{*}},\;{\dot{a}}_{\ell }^{*}=-\frac{1}{i\hbar }\frac{\partial H}{\partial a_{\ell }},\;{\dot{b}}_{\ell }^{*}=-\frac{1}{i\hbar }\frac{\partial H}{\partial b_{\ell }},$$

associated to the effective Hamiltonian:

$$H=\langle \Phi |\hat{H}|\Phi \rangle =H_{a}+H_{b}+W\sum_{i}|a_{i}{|}^{2}{\left|b_{i}\right|}^{2},$$

$$H_{c}=\sum_{i}\left[\frac{U_{c}}{2}{\left|c_{i}\right|}^{4}-J_{c}\left(c_{i}^{*}c_{i+1}+c_{i+1}^{*}c_{i}\right)\right]\;,\;\;\;c=a,b,$$

one obtains by applying the time-dependent variational procedure to model (1) (see, for example, [34]). If the two components are decoupled, namely W=0, one obtains two independent systems in which the uniform configuration featuring:

(4) $$a_{i}\left(t\right)=a_{i}e^{-i\mu_{a}t/\hbar },\;b_{i}\left(t\right)=b_{i}e^{-i\mu_{b}t/\hbar },$$

with $a_{i}=a$ and $b_{i}=b$, can be easily shown to correspond to the ground state, as witnessed by the fact that the effective total energy $\langle \Phi |H_{c}|\Phi \rangle -\mu_{c}N_{c}$ reaches its minimum value. The motion equations are reduced to the equation pair:

$$\mu_{a}=U_{a}{\left|a\right|}^{2}-2J_{a},\;\mu_{b}=U_{b}{\left|b\right|}^{2}-2J_{b},$$

determining $\mu_{a}$, $\mu_{b}$ thanks to the conservation of the boson numbers $N_{a}=L{\left|a\right|}^{2}$, $N_{b}=L{\left|b\right|}^{2}$. For coupled components (W>0), the study of the three-well system has clearly shown how the ground state does not necessarily coincide with the uniform configuration so that, in general, the condition $a_{i}=a$ and $b_{i}=b$ cannot be applied any longer. For W>0, the system:

$$\mu_{a}a_{\ell }=\left(U_{a}|a_{\ell }{|}^{2}+W{\left|b_{\ell }\right|}^{2}\right)a_{\ell }-J_{a}\left(a_{\ell +1}+a_{\ell -1}\right),$$

$$\mu_{b}b_{\ell }=\left(U_{b}|b_{\ell }{|}^{2}+W{\left|a_{\ell }\right|}^{2}\right)b_{\ell }-J_{b}\left(b_{\ell +1}+b_{\ell -1}\right),$$

obtained by substituting the collective-frequency modes (4) in Equations (2) and (3), provides the ground state of the system. Its highly nonlinear character, in general, requires the search of numerical solutions. Interestingly, however, the analysis developed in [30,31] reveals that analytical solutions can be found if one considers the case of population $N_{a}$ and $N_{b}$ to be sufficiently large. This becomes evident by effecting the substitutions $x_{i}=a_{i}/{\sqrt{N}}_{a}$, and $y_{i}=b_{i}/{\sqrt{N}}_{b}$. The previous equations take the form:

(5) $$\lambda_{a}a_{\ell }=\left(\rho_{\ell }+\alpha \beta \eta_{\ell }\right)a_{\ell }-\tau_{a}\left(a_{\ell +1}+a_{\ell -1}\right),$$

(6) $$\lambda_{b}b_{\ell }=\left(\eta_{\ell }+\frac{\alpha }{\beta }\rho_{\ell }\right)b_{\ell }-\tau_{b}\left(b_{\ell +1}+b_{\ell -1}\right),$$

where the local densities $\rho_{\ell }={\left|a_{\ell }\right|}^{2}/N_{a}\leq 1$, and $\eta_{\ell }={\left|b_{\ell }\right|}^{2}/N_{b}\leq 1$ obey the boson-number constraints.

(7) $$1=\sum_{i}\rho_{i},\;1=\sum_{i}\eta_{i},$$

and the effective chemical potentials and hopping amplitudes:

$$\lambda_{a}=\frac{\mu_{a}}{U_{a}N_{a}},\;\;\;\lambda_{b}=\frac{\mu_{b}}{U_{b}N_{b}},\;\tau_{a}=\frac{J_{a}}{U_{a}N_{a}},\;\;\;\tau_{b}=\frac{J_{b}}{U_{b}N_{b}},$$

together with the new parameters:

(8) $$\alpha =\frac{W}{\sqrt{U_{a}U_{b}}},\;\beta =\frac{N_{b}}{N_{a}}\sqrt{\frac{U_{b}}{U_{a}}}.$$

are introduced. Parameters α and β are particularly useful because they embody the significant information about the mixture: α>1 (α<1) distinguishes the strong (weak) interspecies-interaction regime, while β measures the asymmetry of the mixture with respect to the twin-component case β=1 for which $N_{b}=N_{a}$ if $U_{b}=U_{a}$. Note that, in principle, both α and β range in the interval [0,∞]. For β>1, however, one can swap the labels *a* and *b* of the two components, thus, recovering a parameter choice satisfying β≤1. Then, hereafter, the latter inequality is assumed to define the range of β.

For sufficiently large $N_{a}$ and $N_{b}$, one easily sees how the effective amplitudes $\tau_{a}$ and $\tau_{b}$ become negligible quantities so that Equations (5) and (6) reduce to:

(9) $$\lambda_{a}a_{\ell }=\left(\rho_{\ell }+\alpha \beta \eta_{\ell }\right)a_{\ell },\;\;\;\lambda_{b}b_{\ell }=\left(\eta_{\ell }+\frac{\alpha }{\beta }\rho_{\ell }\right)b_{\ell }.$$

This strategy enables one to reconstruct the essential structure of the ground-state phase diagram in which the large-population condition, entailing $\tau_{a},\tau_{b}\to 0$, can be interpreted as a sort of thermodynamic limit according to the statistical-mechanical approach discussed in [36,37]. Its effectiveness is attested by the simple derivation of the phases’ landscape for the three-well system for both repulsive [31] and attractive [33] interspecies interaction. It is worth mentioning a similar version of this scheme, recently applied to study the critical properties of few-fermion systems [38], where the limit $N_{a},N_{b}>>1$ is replaced by the equivalent assumption that intraspecies interactions are sufficiently strong.

## 3. Phase Separation in the BH Dimer

We derive the characteristic phases of the two-well system for $\tau_{a}=\tau_{b}\to 0$ and compare them with those occurring in the three-well phase diagram. The two-well case allows us both to highlight the spatial separation mechanism in its most elementary form, and to exemplify the method we apply to determine the ground-state configurations when α and β are varied. In the two-well case, Equation (9) feature three types of solutions:

$$\left(1\right)\;a_{1},a_{2}\neq 0,\;b_{1},b_{2}\neq 0,$$

$$\left(2\right)\;a_{1},a_{2},b_{2}\neq 0,\;b_{1}=0,$$

$$\left(3\right)\;a_{1},b_{2}\neq 0,\;a_{2},b_{1}=0,$$

which describe fully mixed, partially demixed and fully demixed components, respectively.

*Full-mixing solution*. In the first case, after suppressing factors $a_{\ell }$, and $b_{\ell }$ in system (9), the latter reduces to:

(10) $$\lambda_{a}=\rho_{\ell }+\alpha \beta \eta_{\ell },\;\;\;\lambda_{b}=\eta_{\ell }+\frac{\alpha }{\beta }\rho_{\ell },\;\ell =1,2$$

entailing twin densities for each component:

$$\rho_{1}=\rho_{2}=\frac{\lambda_{a}-\alpha \beta \lambda_{b}}{1-\alpha^{2}},\;\;\;\eta_{1}=\eta_{2}=\frac{\beta \lambda_{b}-\alpha \lambda_{a}}{\beta (1-\alpha^{2})}.$$

In view of this, constraints (7) then impose that:

(11) $$\rho_{1}=\rho_{2}=1/2,\;\eta_{1}=\eta_{2}=1/2,$$

thus providing the conditions whereby unknown parameters $\lambda_{a}$, $\lambda_{b}$ can be fixed. One obtains $\lambda_{a}=\left(1+\alpha \beta \right)/2$ and $\lambda_{b}=\left(1+\alpha /\beta \right)/2$. Equation (11) describe the well-known uniform solution which exists for any value of α and β but loses its status as the ground state for α>1. In the large-population limit, this inequality in fact coincides with the condition α>1+2τ→1 (twin components with $\tau_{a}=\tau_{b}=\tau$ were assumed) for which the perturbation of the uniform solution within the Bogoliubov scheme develops unstable oscillations [23,39] and then can no longer represent the ground state. The second-type and third-type solutions, however, offer alternative candidates for the ground-state configuration.

*Single-well-mixing solution*. In the second case, the assumption that component *b* is absent in the second well ($y_{2}=0\to \eta_{2}=0$), so that only the first well features mixing, implies that:

$$\lambda_{a}=\rho_{1}+\alpha \beta \eta_{1},\;\lambda_{a}=\rho_{2},\;\lambda_{b}=\eta_{1}+\frac{\alpha }{\beta }\rho_{1},$$

with the constraints $1=\rho_{1}+\rho_{2}$ and $1=\eta_{1}$. The solution is then given by

(12) $$\rho_{1}=\frac{1-\alpha \beta }{2},\;\;\rho_{2}=\frac{1+\alpha \beta }{2},\;\eta_{1}=1,\;\;\eta_{2}=0,$$

showing how the increasing demixing corresponds to a residual single-well-mixing (SWM) in one of the two wells. Of course, a perfectly equivalent solution is obtained by assuming $b_{1}=0$ in place of $b_{2}=0$ which simply entails the exchange of the analytic expressions describing $\rho_{2}$ and $\rho_{1}$. The crucial information emerging from solution (12) is that the relevant definition domain must satisfy the inequality β<1/α to prevent a negative density $\rho_{1}$.

*Full-demixing solution*. Finally, we consider the third case $a_{2}=0=b_{1}$, $a_{1}=1=b_{2}$, which features complete space separation. Imposing the constraints (7) gives:

(13) $$\rho_{1}=1,\;\;\rho_{2}=0,\;\eta_{1}=0,\;\;\eta_{2}=1.$$

As a consequence, system (9) is easily satisfied in that the equations for $a_{2}$ and $b_{1}$ are simply eliminated, while the remaining two equations $\lambda_{a}=\rho_{1}\equiv 1$, $\lambda_{b}=\eta_{2}\equiv 1$ serve to fix the effective chemical potentials.

**Figure 1:**
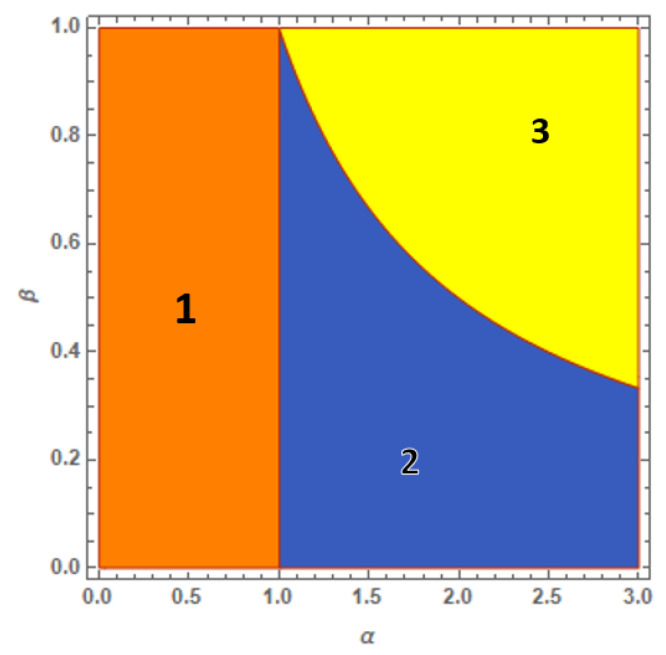
(Color Online): phase diagram of the BH dimer. The uniform solution corresponds to region 1 where α<1. Region 2 is associated to the partially mixed phase which features a well with the minority component mixed with a fraction of the majority component and the second well with the remaining fraction of the majority component. Region 3 corresponds to the fully demixed solution with β≥1/α.

The resulting scenario is summarized in Figure 1, where regions 1, 2 and 3 refer to ground-state phases associated to solutions (11)–(13), respectively. The comparison of the three solutions shows that, while SWM solution (12) coalesces with solution (13) along the hyperbole β=1/α, solution (12) does not match solution (11) for α=1, thus revealing a discontinuous behavior.

Inequality α<1, rewritten as $W<\sqrt{U_{a}U_{b}}$, shows how region 1 represents the weak-interaction regime in which the inter-species repulsion is too small to cause spatial separation. However, the possibility to directly reach region 3 with fully demixed components is given only for β=1 when $N_{b}/N_{a}=\sqrt{U_{a}/U_{b}}$. In any other case, β<1, the crossing of region 2, the intermediate partial-mixing phase, represents the unavoidable process whereby the residual SWM is suppressed by increasing α and region 3 is then reached. This circumstance highlights the important role of the asymmetry degree of the system which, through the inequality β>1/α, determines the critical value $W=\sqrt{U_{a}U_{b}}/\beta$ for which the interaction is strong enough to trigger full demixing.

The ground-state map, namely, the phase diagram in Figure 1, is derived by combining the knowledge of the energies corresponding to each solution with the information about their definition domains. These energies read (${\bar{E}}_{\sigma }=E_{\sigma }/U_{a}N_{a}^{2}$, σ=1,2,3):

$${\bar{E}}_{1}=\frac{1}{4}\left(1+\beta^{2}+2\alpha \beta \right),$$

$${\bar{E}}_{2}=\frac{1}{4}\left(1+2\beta^{2}+2\alpha \beta -\alpha^{2}\beta^{2}\right),\;{\bar{E}}_{3}=\frac{1}{4}\left(1+\beta^{2}\right).$$

One easily checks that:

$${\bar{E}}_{1}\equiv {\bar{E}}_{2},\;\;\mathrm{for}\;\;\alpha =1,\;{\bar{E}}_{2}\equiv {\bar{E}}_{3},\;\;\mathrm{for}\;\;\beta =\frac{1}{\alpha }.$$

More interestingly, one finds ${\bar{E}}_{1}<{\bar{E}}_{2}$ for α<1, while for α>1 one has ${\bar{E}}_{1}>{\bar{E}}_{2}$. Then, the ground-state status is transferred to solution (12) for α>1. This property is confirmed, in parallel, by the fact that the inequality:

$${\bar{E}}_{2}<{\bar{E}}_{3},\;D_{2}=\left\{\left(\alpha ,\beta \right):\alpha >1,\beta <1/\alpha \right\},$$

is always valid in $D_{2}$ (where both solutions (12) and (13) are well defined), then excluding the possibility that solution (13) is the ground state. The latter inherits this role in $D_{3}=\left\{\left(\alpha ,\beta \right):\beta <1,\beta >1/\alpha \right\}$ (region 3) where ${\bar{E}}_{3}$ is the minimum energy.

### Comparison with the BH Trimer

**Figure 2:**
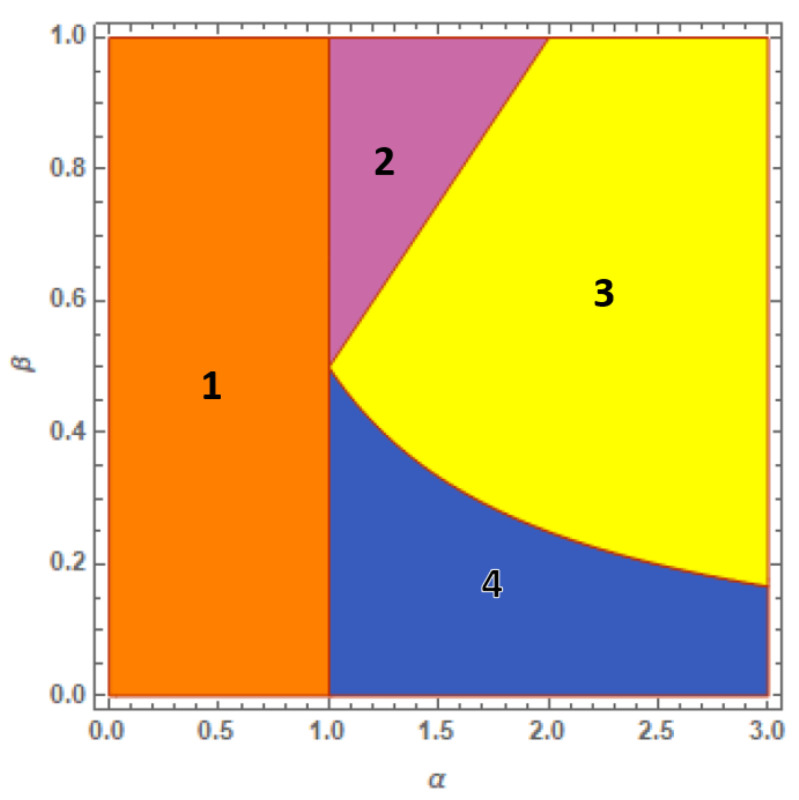
(Color Online) Phase diagram of the 3-well system in the limit $\tau_{a},\tau_{b}\to 0$. Phases 4 and 2 (partial-demixing solutions (15) and (16)) separate phase 1 (uniform solution) and phase 3 (full-demixing solutions (14)). The comparison with the 2-well phase diagram in Figure 1 shows how the presence of the third well causes the onset of phase 2, representing a second independent way to realize partial demixing.

The ground-state phase diagram of the BH trimer is illustrated in Figure 2 and includes four phases. These correspond to the definition domains of four different solutions to Equation (9) which represent the lowest energy configurations. Their derivation, discussed in Ref. [31], involves six local densities since ℓ=1,2,3.

Phase 1 corresponds to the uniform solution characterized by full mixing and the local densities $\rho_{\ell }=\eta_{\ell }=1/3$, with ℓ=1,2,3, of the two components among the three wells. As for the two-well system, the border α=1 of phase 1 coincides with the upper limit of the weak interaction regime: for α<1, the inter-species repulsion is too small to cause spatial separation.

Phase 3 in Figure 2 represents the opposite regime characterized by complete demixing: one species clots in one site, while the other spreads the remaining two. One has, for example:

(14) $$\rho_{1}=0,\;\;\rho_{2}=\rho_{3}=\frac{1}{2},\;\eta_{1}=1,\;\;\eta_{2}=\eta_{3}=0.$$

This phase is delimited by 1/α<β<α/2. It occurs for values of α sufficiently large and includes both symmetric (β=1) and asymmetric components (0<β<1). Phase 3 shows how, except for β=1/2, the transition from the fully mixed phase 1 to the fully demixed phase 3 cannot occur without the crossing of intermediate phases (either 2 or 4) in which mixing is progressively suppressed.

Phases 2 and 4 intercalate phases 1 and 3. Phase 4, delimited by α>1 and β<1/(2α), is quite naturally associated to the phase 2 of Figure 1. Partial demixing of phase 4 exhibits the same space structure of the SWM solution (12): The minority component clots in a single site together with a small fraction of the majority component, however, the remaining fraction of the majority component occupies the other two wells. This is exemplified by the solution:

(15) $$\left\{\begin{array}{c} \rho_{1}=\frac{1-2\alpha \beta }{3},\rho_{2}=\rho_{3}=\frac{1+\alpha \beta }{3},\\ \eta_{1}=1,\;\;\eta_{2}=\eta_{3}=0. \end{array}\right.$$

The symmetry of Equation (9) under simple permutations of the local densities shows how, altogether, one obtains three, six and three equivalent realizations of a given solution to (14)–(16), respectively.

Phase 2 gives the possibility to follow a second independent path to reach phase 3 from phase 1. It exhibits partial demixing, namely the ground-state solution features two wells with complete demixing (the two components occupy different wells) while the remaining fractions of the two components are mixed in the third well:

(16) $$\left\{\begin{array}{c} \rho_{1}=0,\;\;\rho_{2}=\frac{\alpha^{2}-\alpha \beta -2}{\alpha^{2}-4},\;\;\rho_{3}=1-\rho_{2}\;,\\ \eta_{1}=\frac{\alpha^{2}\beta -\alpha -2\beta }{(\alpha^{2}-4)\beta },\;\;\eta_{2}=0,\;\;\eta_{3}=1-\eta_{1}\;. \end{array}\right.$$

This phase is delimited by 1<α<2β, thus corresponding to intermediate values of the effective interaction α and not overly asymmetric components (1/2<β<1).

As observed above, phase 4 is essentially equivalent to phase 2 in Figure 1 due to the similarity of the relevant SWM solutions (12) and (15), and of the hyperbolic curve constituting their upper phase boundary. The significant change exhibited by the trimer phase diagram is thus the appearance of phase 2 for 1/2<β<1, a ground-state solution absent in the dimer phase diagram. The presence of the third well triggers, for 1/2<β<1, a parallel mechanism to reach the full-demixing regime in which the reduction in mixing coexists with the fact that each component is distributed on at least two of three wells. This mechanism significantly modifies for β<1/2 when, similar to the dimer behavior, the minority component is confined in a single well.

## 4. Phase Diagram of the Four-Well System

**Figure 3:**
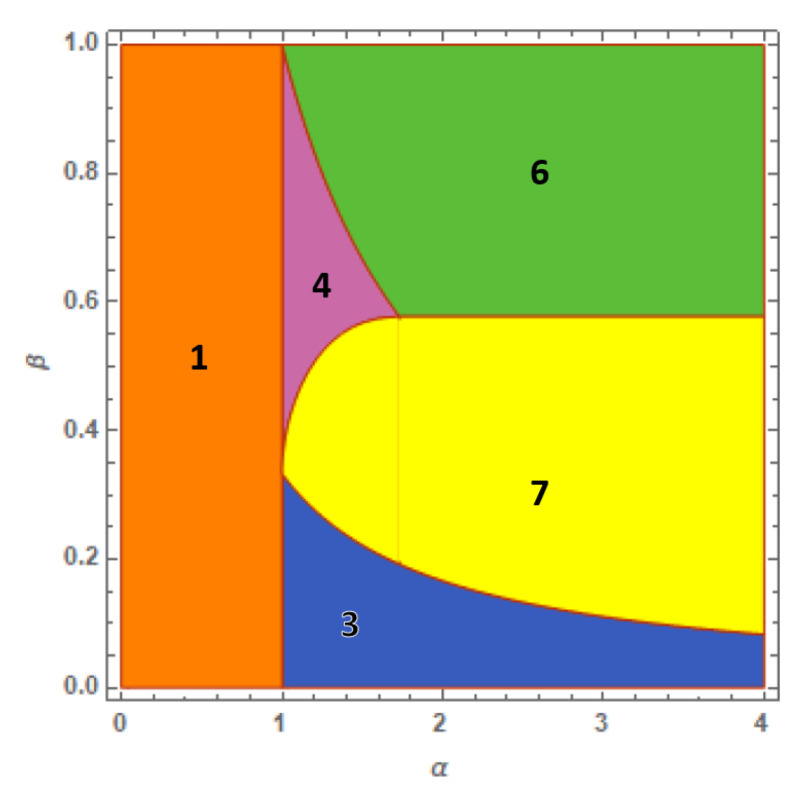
(Color Online) Phase diagram of the 4-well system in the limit $\tau_{a},\tau_{b}\to 0$. Phase 1 (uniform solution (17)) and phases 6 and 7 (full-demixing solutions (20) and (21)) are intercalated by phases 3 and 4 (partial-demixing solutions (18) and (19)) which highlight two different ways to enact spatial phase separation. The comparison with Figure 2 shows how the presence of the fourth well triggers the onset of phases 6 and 7.

When the ring lattice is formed by four wells, the equation system (9) depends on eight local densities $\rho_{\ell }$ and $\eta_{\ell }$ with ℓ∈[1,4]. The number of solutions considerably increases and before reconstructing the ground-state map, one must identify the set of solutions which cannot represent the ground state whilst representing excited states. For this reason, below we focus our attention on the four solutions which, in addition to the expected uniform solution, result in the formation of the four-well phase diagram. Details about their derivation and the presence of other solutions not fitting the minimum-energy condition are discussed in Appendix A. To better highlight the structure of the phase diagram described in Figure 3 we explicitly mention, at each step, the link between phases and solutions.

*Fully mixed solution*. This is the uniform solution, which corresponds to phase 1, and is obtained from configuration (A1). It exhibits the usual form:

(17) $$\rho_{\ell }=1/4,\;\eta_{\ell }=1/4,\;\forall \ell .$$

*SWM solution*. This solution describes phase 3. The space distribution obtained from configuration (A3) predicts that the minority component clots in a single well where a small fraction of the majority component survives causing residual mixing:

(18) $$\left\{\begin{array}{c} \rho_{1}=\frac{1-3\alpha \beta }{4},\;\rho_{k}=\frac{1+\alpha \beta }{4},\\ \eta_{1}=1,\;\;\eta_{k}=0,\;k=2,3,4. \end{array}\right.$$

This configuration, defined for:

$$\beta <\frac{1}{3\alpha },$$

also characterizes both the two-well (see solution (12)) and the three-well cases (see solution (15)). As discussed in the three-well case, the complete confinement of one component in a single well allows one to regard (18) as the configuration that, before reaching full demixing, features the most pronounced demixing degree.

*Weak-demixing solution*. Phase 4 is described by the space distribution ensuing from the trial solution (A4). The distribution has the form:

(19) $$\left\{\begin{array}{c} \rho_{1}=\rho_{3}=\frac{1+\alpha \beta }{4},\;\;\rho_{2}=\rho_{4}=\frac{1-\alpha \beta }{4},\\ \eta_{2}=\eta_{4}=\frac{1}{2},\;\;\eta_{1}=\eta_{3}=0, \end{array}\right.$$

and is defined for β<1/α. It describes a double-dimer structure in which the distribution of the two components characterizing wells 1 and 2 is equal to the distribution in the wells 3 and 4 and corresponds to the dimer solution (12). Such a state, by definition, cannot occur in a system with three wells. Note that going from the two-well to the four-well case highlights how the SWM solution (12) (phase 2 in Figure 1) is now replaced by two different intermediate configurations (solutions (18) and (19)) instead of one due to the larger number of wells. They exhibit different realization of partial demixing.

*Twin-dimer full-demixing solution*. This is associated to phase 6 and is obtained from configuration (A6). The relevant components’ distribution reads:

(20) $$\left\{\begin{array}{c} \rho_{1}=0,\;\;\rho_{2}=\frac{1}{2},\;\;\eta_{1}=\frac{1}{2},\;\;\eta_{2}=0,\\ \rho_{3}=0,\;\;\rho_{4}=\frac{1}{2},\;\;\eta_{3}=\frac{1}{2},\;\;\eta_{4}=0. \end{array}\right.$$

It essentially represents the fully demixed solutions (see solution (13)) for a pair of twin two-well systems.

*Full-demixing asymmetric state*. Solution (A7) entails the asymmetric distribution (associated to phase 7):

(21) $$\rho_{1}=0,\;\rho_{k}=\frac{1}{3},\;\eta_{1}=1,\;\eta_{k}=0,\;k=2,3,4.$$

As for the trimer phase diagram of Figure 2, the four-well phase diagram described in Figure 3 shows how the fully mixed phase 1 (uniform solution) and the full-demixing domain, formed by phase 6 (solution (20)) and phase 7 (solution (21)), are intercalated by phase 3 (solution (18)) and phase 4 (solution (19)), exhibiting partial demixing.

*Other solutions*. The equation system (9) provides two further solutions that are physically meaningful and are characterized by partial demixing. These are described by formulas (A9) and (A12) in Appendix A. However, the comparison of their energies with those corresponding to the previous solutions reveals how they represent in any case excited states, and thus cannot be associated to any phase in the four-well phase diagram.

### 4.1. Phase Diagram Derivation and Characteristic Ground-State Energies

The ground-state energies ${\bar{E}}_{k}=E_{k}/\left(U_{a}N_{a}^{2}\right)$ (index *k* corresponds to the phase index) of the five phases in Figure 3 are easily obtained by substituting the corresponding expressions of the local densities in the four-well Hamiltonian with $\tau_{a}=\tau_{b}=0$. The boundaries of each phase are recognized by determining the domain in which, for a given *k*, the condition ${\bar{E}}_{k}\leq {\bar{E}}_{i}$ is verified for any other i≠k. As for the dimer case, this information combined with the definition domains of each solution allows one to reconstruct the phase boundaries visible in Figure 3.

The results obtained by applying this procedure are summarized in the following list which describes the ground-state energies and the relevant definition domains:

$${\bar{E}}_{1}=\frac{1+\beta^{2}+2\alpha \beta }{8}\;\left(\alpha <1,\;\;\beta <1\right),\;{\bar{E}}_{3}=\frac{1+2\alpha \beta +\beta^{2}\left(4-3\alpha^{2}\right)}{8}\;\left(\alpha >1,\;\;\beta <\frac{1}{3\alpha }\right),$$

$${\bar{E}}_{4}=\frac{1+2\alpha \beta +\beta^{2}\left(2-\alpha^{2}\right)}{8}\;\;\left(\alpha >1,\;\;\;f\left(\alpha \right)<\beta <\frac{1}{\alpha }\right),\;{\bar{E}}_{6}=\frac{1+\beta^{2}}{4}\;\;\left(\left(\alpha ,\beta \right)\in D_{6}\cup D_{6}^{\prime }\right),$$

with $D_{6}=\left\{\alpha \in \left[1,\sqrt{3}\right]:1/\alpha <\beta <1\right\}$ and $D_{6}^{\prime }=\left\{\alpha >\sqrt{3}:1/\sqrt{3}<\beta <1\right\}$, and:

$${\bar{E}}_{7}=\frac{1+3\beta^{2}}{6}\;\left(\left(\alpha ,\beta \right)\in D_{7}=\left\{\frac{1}{3\alpha }<\beta <F\left(\alpha \right),\;\alpha \in \left[1,\infty \right]\right\}\right),$$

with:

$$f\left(\alpha \right)=\frac{\alpha +\sqrt{\frac{2}{3}\left(\alpha^{2}-1\right)}}{2+\alpha^{2}},\;\;F\left(\alpha \right)=\left\{\begin{array}{c} \;f\left(\alpha \right),\;\;\alpha \in \left[1,\sqrt{3}\right],\\ \;\frac{1}{\sqrt{3}},\;\;\alpha \in \left[\sqrt{3},\infty \right]. \end{array}\right.$$

**Figure 4:**
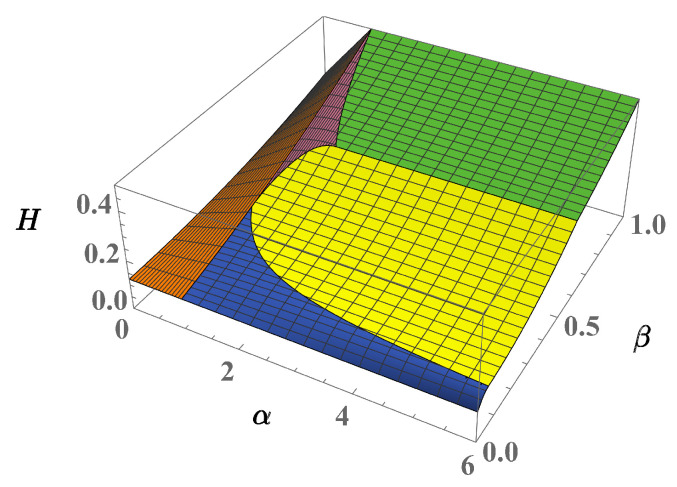
(Color Online) Energy landscape corresponding to the phase diagram of the 4-well system in the limit $\tau_{a},\tau_{b}\to 0$. Vertical axis: *H* describes the ground-state effective energy ${\bar{E}}_{k}=E_{k}/\left(U_{a}N_{a}^{2}\right)$.

The phase diagram shown in Figure 4 displays the ground-state domains corresponding to this energy scenario. For the sake of clarity, we briefly sketch the essential steps that allows one to reconstruct the four-well phase diagram. One begins by considering the uniform solution (17) which identifies with the ground state for α<1 since one easily shows that ${\bar{E}}_{1}<{\bar{E}}_{k}$ for any k≠1. Then, the fact that ${\bar{E}}_{1}={\bar{E}}_{3}={\bar{E}}_{4}$, for α=1, suggests that phases 3 and 4 associated to solutions (18) and (19), respectively, could be the ground state for α>1. Concerning phase 3, solutions (18) is confirmed to be the ground state because inequalities ${\bar{E}}_{3}<{\bar{E}}_{4},{\bar{E}}_{6},{\bar{E}}_{7}$ are verified in the domain α>1, and β<1/(3α). In parallel, the validity domains of inequalities ${\bar{E}}_{4}<{\bar{E}}_{6}$ and ${\bar{E}}_{4}<{\bar{E}}_{7}$ are found to be:

β<1/α,β<f(α)

respectively, which highlights, together with α>1, the definition domain of phase 4 in which solution (19) is the ground state. Note that the two boundaries β=1/α and β=f(α) (implicitly defined by the previous inequalities) are such that $1/\alpha =f\left(\alpha \right)=1/\sqrt{3}$ for $\alpha =\sqrt{3}$. The value $\beta =1/\sqrt{3}$ has a second important role: it represents the straight line separating phases 7 and 6. This clearly emerges from inequality ${\bar{E}}_{7}<{\bar{E}}_{6}$ whose validity domain is described by $\beta <1/\sqrt{3}$. The recognition of the definition domains in which $E_{6}$ and $E_{7}$ are the ground-state energies completes the phase diagram exploration.

### 4.2. Discussion

The distinctive property of the four-well phase diagram is the presence of two independent realizations of the full-demixing state associated to solution (20) (phase 6) and solution (21) (phase 7). As a consequence, unlike the three-well phase diagram (Figure 2) where the unique full-demixing regime (phase 3) is reached through phases 2 and 4 for a sufficiently large α, the suppression of mixing in the four-well case can be realized in three independent ways (intuitively described by the phase sequences 1–4–6, 1–4–7 and 1–3–7) leading to the full-demixing phases 6 and 7. More specifically, the emergence of phase 6 significantly modifies the structure of the four-well phase diagram which otherwise would display a structure, with four domains, qualitatively similar to that of the three-well phase diagram for $\beta <1/\sqrt{3}$.

A common trait with the two-well and three-well phase diagrams emerges for a sufficiently small β. Similarly to the three-well case for β<1/2, the four-well phase diagram reproduces, for β<1/3, the structure of the domain in the two-well phase diagram corresponding to the SWM solution (phase 2). This circumstance suggests that this behavior (and the relevant SWM solution) should also persist for a higher number of wells and sufficiently small β. Note that, for $U_{a}=U_{b}$, the inequalities defining the upper boundaries β<1/α, β<1/2α, and β<1/3α, of strong-demixing solutions (12), (15) and (18), respectively, become:

$$\frac{N_{b}}{N_{a}}<\frac{1}{\alpha },\;\frac{N_{b}}{N_{a}}<\frac{1}{2\alpha },\;\frac{N_{b}}{N_{a}}<\frac{1}{3\alpha },$$

entailing that, for a given value of α, the access to the lower SWM phase in the three phase diagrams requires that $N_{b}$ is proportional to a decreasing fraction 1/(L−1) of $N_{a}$ when the well-number *L* is increased.

Another aspect characterizing the four-well phase diagram is the behavior of the local densities at the phase boundaries separating phases *ℓ* and *k* that we denote with $\gamma_{\ell k}$. Considering solutions (17)–(21), one easily discovers that the phase-*ℓ* solution and the phase-*k* solution match each other only along $\gamma_{37}$ and $\gamma_{46}$, whereas the ground state exhibits a discontinuous behavior of the local densities when crossing $\gamma_{14}$, $\gamma_{13}$, $\gamma_{47}$, and $\gamma_{67}$.

The discontinuities observed when going from the full-mixing phase 1 to partial-demixing phases (boundaries $\gamma_{14}$, $\gamma_{13}$ at α=1) represents a common feature with the two-well and three-well phase diagrams as well as the continuous character of local densities when crossing the upper boundaries β=1/[(L−1)α] of the SWM-solution phases for L=2,3,4. Unlike boundaries $\gamma_{47}$, and $\gamma_{67}$ of the four-well case, one easily checks that the two-well and three-well phase diagrams do not feature further phase boundaries affected by local-density discontinuity for α>1.

**Figure 5:**
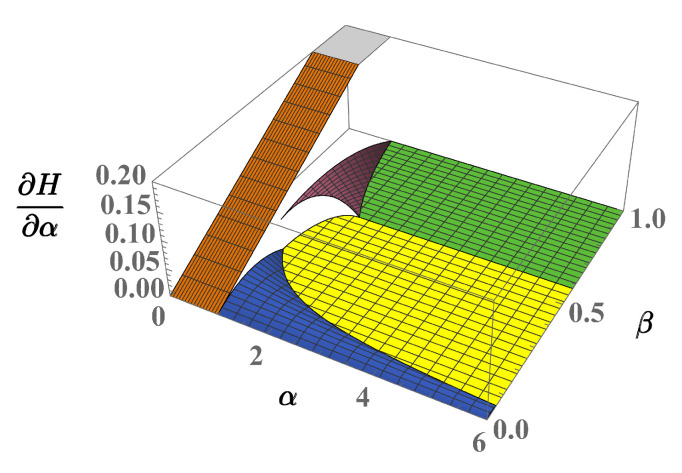
(Color Online) Discontinuities of effective energy $H={\bar{E}}_{k}$ with respect to parameter α. Vertical axis: $\partial_{\alpha }H$ describes the first derivative of *H*.

Interestingly, this feature can be put in relation to the well-known property of thermodynamic potentials that links the presence of a transition to the discontinuities of the partial derivatives of potentials. In the current case, the free energy F=E−TS=E (at temperature T=0) and its partial derivatives allow one to detect possible critical behaviors at the phase boundaries $\gamma_{k\ell }$. Despite the continuous character of energy $\bar{E}=$$E/(U_{a}N_{a}^{2})$, well illustrated in Figure 4, one easily discovers that at least one of the following discontinuity conditions:

$$\partial_{\alpha }{\bar{E}}_{k}\neq \partial_{\alpha }{\bar{E}}_{\ell },\;\partial_{\beta }{\bar{E}}_{k}\neq \partial_{\beta }{\bar{E}}_{\ell },$$

involving first-order derivatives takes place for any pair (k,ℓ) except for (k,ℓ)=(3,7),(4,6) (see Figure 5). Concerning the latter cases, however, their continuity is lost when considering second-order derivatives.

Instead of representing the behavior of the *F* derivatives we confirm the presence of the critical behavior by exploiting two indicators of spatial order which, in the case of the three-well systems, were proven to effectively detect the different domains of the phase diagram [31,33]. These are [40,41], the mixing entropy and the location entropy:

$$S_{mix}=-\frac{1}{2}\sum_{i=1}^{L}\left(\rho_{i}\ln\frac{\rho_{i}}{\rho_{i}+\eta_{i}}+\eta_{i}\ln\frac{\eta_{i}}{\rho_{i}+\eta_{i}}\right),\;S_{loc}=-\sum_{i=1}^{L}\frac{\rho_{i}+\eta_{i}}{2}\ln\frac{\rho_{i}+\eta_{i}}{2},$$

respectively. The states of the two-well phase diagram well exemplifies the information provided by $S_{mix}$ and $S_{loc}$. Considering solutions (11)–(13), relevant to the full-mixing, partial-demixing and full-demixing regimes, one finds:

$$S_{mix}=\ln2,\;S_{mix}\neq 0,\;S_{mix}=0,$$

respectively, where ln2 is the maximum value of the mixing entropy. As expected, the maximum and minimum mixing degrees are thus associated to the full-mixing and full-demixing regimes, respectively, while partial demixing corresponds to the intermediate value $S_{mix}\neq 0$. Location entropy $S_{loc}$ measures the dispersion of the two components in the two wells. The dispersion reaches its minimum value if both components are localized in the same well. For example, by setting $\rho_{1}=\eta_{1}=1$, $\rho_{2}=\eta_{2}=0$ m one obtains $S_{loc}=0$. Conversely, the full-mixing state (11) entails maximum dispersion $S_{loc}=\ln2$ while state (13) gives the intermediate value $S_{loc}=\ln2/2$.

**Figure 6:**
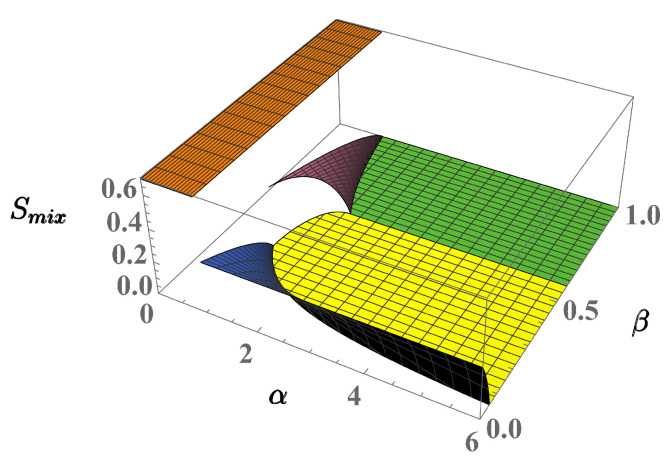
(Color Online) Mixing entropy of the 4-well system in the limit $\tau_{a},\tau_{b}\to 0$.

**Figure 7:**
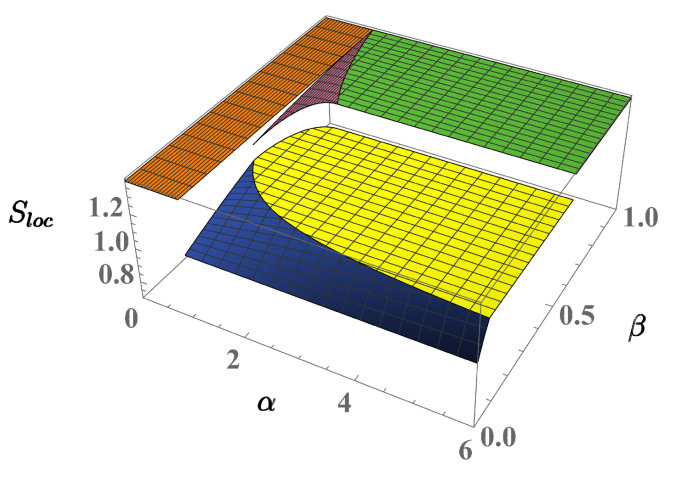
(Color Online) Location entropy of the 4-well system in the limit $\tau_{a},\tau_{b}\to 0$.

As applied to the four-well phase diagram, the behavior of $S_{mix}$, described in Figure 6 clearly shows the discontinuity characterizing the transition from the fully mixed state to the partial-demixing states (phases 3 and 4), when crossing boundaries $\gamma_{13}$ and $\gamma_{14}$. This indicator also captures the discontinuity relevant to the transition from phase 4 to phase 7 (boundary $\gamma_{47}$), but fails to detect the discontinuity between phases 6 and 7. The latter and the relevant boundaries $\gamma_{67}$ are successfully captured by $S_{loc}$, as shown in Figure 7, which is able to distinguish the different dispersion degree for the two full-demixing states. Quite consistently, neither $S_{mix}$ nor $S_{loc}$ exhibit discontinuous behaviors at $\gamma_{37}$ and $\gamma_{46}$ where, despite the crossing, the local densities of both components remain continuous.

*Finite-size effects*. The study of mixtures in the ring trimer [31,33] has clearly shown how the information obtained in the limit τ→0 about the three-well phase diagram (Figure 2) and its characteristic phases is still qualitatively valid when considering finite-size effects, namely for small but non-zero τ (finite boson populations).

If τ is small, the local-density solutions satisfy Equations (5) and (6) and the jump discontinuities affecting solutions (17)–(21) at certain boundaries $\gamma_{\ell k}$ are removed, substituted by smooth junctions embodying the continuous dependence of local densities from α and β. This reflects, of course, on the jump discontinuities exhibited by $S_{mix}$ and $S_{loc}$ (function of local densities) which are replaced by rapid but continuous changes inside restricted thin domains. For τ→0, these domains collapse to the original boundaries $\gamma_{\ell k}$, while jump discontinuities of local densities crop up at phase boundaries. In this scenario, the phases of the large-population limit remain well recognizable, confirming the fact that the phase diagrams of Figure 1, Figure 2 and Figure 3 incorporate the essential information on the ground-state properties of *L*-well systems.

*Conjectures for large-size rings*. One can show that certain ground-state configurations suggested by the small-size ring analysis can be extended to rings with a large well number *L*. To this end, it is useful to remember that ring lattices are distinguished by the fact that *L* is either even or odd.

Let us first consider the case with odd L=2s+1, s∈N. We conjecture that, when β is sufficiently close to β=1, there exists an intermediate partial-demixing phase with a solution exhibiting the same structure of solution (16) (associated to phase 2 in Figure 2) exhibiting almost complete demixing except for a single well. For the sake of simplicity, we assume that the residual mixing occurs at a single well, j=s+1, so that only $\rho_{s+1}$ and $\eta_{s+1}$ are simultaneously non-zero. This amounts to setting:

(22) $$\rho_{r}\neq 0,\;\;\eta_{r}=0,\;\;r\in \left[1,s\right],\;\rho_{r}=0,\;\;\eta_{r}\neq 0,\;\;r\in \left[s+2,2s+1\right],$$

$$\rho_{s+1}\neq 0,\;\eta_{s+1}\neq 0.$$

With this assumption, solving Equation (9) gives $\rho_{1}=\rho_{2}=\cdots =\rho_{s}$, and $\eta_{s+2}=\eta_{s+3}=\cdots =\eta_{L}$ with:

(23) $$\left\{\begin{array}{cc} \; & \rho_{1}=\frac{\alpha \beta -s\alpha^{2}+s+1}{{\left(1+s\right)}^{2}-\alpha^{2}s^{2}},\;\rho_{s+1}=\frac{1+s-\alpha \beta s}{{\left(1+s\right)}^{2}-\alpha^{2}s^{2}},\\ \; & \eta_{s+1}=\frac{\beta (s+1)-s\alpha }{\beta [{\left(1+s\right)}^{2}-\alpha^{2}s^{2}]},\;\eta_{L}=\frac{\beta \left(s+1\right)+\alpha -s\alpha^{2}\beta }{\beta [{\left(1+s\right)}^{2}-\alpha^{2}s^{2}]}, \end{array}\right.$$

(remember that L=2s+1). One easily checks that the two constraints $s\rho_{1}+\rho_{s+1}=1$ and $\eta_{s+1}+s\eta_{L}=1$ are satisfied. The definition domain is given by

$$1\leq \alpha \leq \frac{s+1}{s},\;\beta \leq 1,\;\beta \geq \frac{\alpha s}{1+s},$$

which extends the definition domain of solution (16) to L=2s+1>3. As for the trimer case, the previous solution exhibits a discontinuous behavior at the border α=1 where it does not match the uniform solution $\rho_{j}=1/L$, $\eta_{j}=1/L$, ∀j. Conversely, the energy is expected to be continuous, a behavior confirmed by the fact that the uniform-solution energy:

$${\bar{E}}_{0}=\frac{1}{2}\left[\left(2s+1\right)\rho_{1}^{2}+\beta^{2}\left(2s+1\right)\eta_{1}^{2}+2\alpha \beta \left(2s+1\right)\rho_{1}\eta_{1}\right],$$

and the solution with the single-well residual mixing (Equation (23)):

$${\bar{E}}_{0}^{\prime }=\frac{1}{2}\left[s\rho_{1}^{2}+\rho_{s+1}^{2}+\beta^{2}\left(s\eta_{L}^{2}+\eta_{s+1}^{2}\right)+2\alpha \beta \rho_{s+1}\eta_{s+1}\right],$$

give, for α=1:

$${\bar{E}}_{0}={\bar{E}}_{0}^{\prime }=\frac{1}{2L}{\left(1+\beta \right)}^{2}.$$

This proves that the two phases merge at the phase-separation line α=1. On the other hand, for β=αs/(1+s) (this curve is the (2s+1)-well version of the boundary separating phases 2 and 3 in Figure 2 for L=3↔s=1) one finds:

$$\rho_{r}=\frac{1}{1+s},\;\;\eta_{r}=0,\;\;r\leq s+1,\;\rho_{r}=0,\;\;\eta_{r}=\frac{1}{s},\;\;r\in \left[s+2,L\right],$$

showing how the current solution characterized by L=2s+1 at the lower border β=αs/(1+s) reproduces the full-demixing solution one expects to find in the phase diagram for a sufficiently large α (this corresponds to the transition from phase 2 to phase 3 in Figure 2).

With even *L*, the weak-demixing solution (19) (associated to phase 4 in Figure 3) of the case L=4 can be easily generalized to case L=2s. One determines:

(24) $$\rho_{2p-1}=\frac{1+\alpha \beta }{2s},\;\;\rho_{2p}=\frac{1-\alpha \beta }{2s},\;\;\eta_{2p}=\frac{1}{s},\;\;\eta_{2p-1}=0,\;p\in \left[1,s\right],$$

(defined in 1<α≤1/β, β≤1) which can be also seen as the *s*-fold replica of solution (12) for the elementary case L=2 (well occupied by the majority component and the remaining well with residual mixing). For β→1/α, one recovers the full-demixing state with $\rho_{2p}=0=\eta_{2p-1}$ while for α→1, the energy of this solution can be shown to match the energy ${\bar{E}}_{0}$ of the full-mixing uniform state. The problem is that this solution (as well as that described by Equation (23) for odd *L*) now represents one among many other (unknown) ground-state solutions that must be identified and organized in order to reconstruct (as shown for the case L=4) the map of adjacent phases forming the phase diagram. This task results to be extremely difficult.

The degree of complexity of the phase diagram for arbitrary *L* can be understood by considering the full-demixing phases in the large-α regime. As suggested by the case L=4 (see phases 6 and 7 in Figure 3), these phases are expected to correspond to a number, increasing with *L*, of different realizations of the full-demixing state. In this case, the ring is essentially formed by *p* wells occupied by one component and L−p wells occupied by the remaining one (cases p=L and p=0 are of course excluded). Then, one has L−2 realizations of demixing:

(25) $$\rho_{1}=\cdots =\rho_{p}=\frac{1}{p},\;\eta_{p+1}=\cdots =\eta_{L}=\frac{1}{L-p},$$

with $\rho_{i}=0$ for i∈[p+1,L] and $\eta_{k}=0$ for k∈[1,p]. This configuration solves Equation (9). Interestingly, the value of *p* can be shown to depend on the value of β by imposing the minimum-energy condition. The energy of the previous configuration:

$$\tilde{E}=\frac{1}{2}\left(\frac{1}{p}+\frac{\beta^{2}}{L-p}\right)$$

entails that $d\tilde{E}/dp=0$ for p=L/(1+β). The number of wells associated to the two components is thus linked to the asymmetry parameter β. For $U_{a}=U_{b}$ and interpreting *p* as an almost continuous parameter, one has $\beta =N_{b}/N_{a}$ with:

(26) $$p=L\frac{N_{a}}{N},\;L-p=L\frac{N_{b}}{N},\;N=N_{a}+N_{b},$$

determining the numbers of wells associated to the two components in the ring lattice. The solutions emerging from the previous discussion for the two cases with even or odd *L* (and β close enough to β=1) and the large-α full-demixing states (25) represent some of the few cases in which one can make a simple conjecture about the structure of the phase diagram in the limit of large *L*, in addition to (1) the uniform solution (and the relevant phase) defined for α≤1 and for any *L*, and (2) the phase represented by solutions (12), (15) and (18) for L=2,3,4, respectively, in the opposite region with sufficiently small β, whose general form for an arbitrary *L*, can be easily shown to be:

(27) $$\rho_{1}=\frac{1-(L-1)\alpha \beta }{L},\;\rho_{2}=\cdots =\rho_{L}=\frac{1+\alpha \beta }{L},\;\eta_{1}=1,\;\eta_{2}=\cdots =\eta_{L}=0.$$

For an arbitrary *L*, the solutions and the complex architecture of the corresponding phases occurring at intermediate values of β and α cannot be predicted in a simple way. Intuitively, one expects to find an increasing number of partial-demixing phases occupying the intermediate sector of the phase diagram which connects the full-mixing uniform phase (α<1) and the full-demixing phases characterized by Equation (26) for a sufficiently large α.

## 5. Conclusions

We reformulated the BH-like model describing the binary mixture distributed in a four-well ring within the coherent-state variational picture in which a set of local order parameters describes the microscopic state of the system, and more specifically, the local populations of the two components. This well-known semiclassical approach allows one to derive the ground-state equations and thus reproduce the mechanism governing the formation of the new phases starting from the weak-interaction regime in which the system is fully mixed and delocalized. As discussed in Section 1, the realization of *L*-well rings, and then the study of their ground-state phases, is within the reach of current trapping techniques [18,19,20]. Various experimental aspects concerning a mixture in a three-well potential (realized by means of tweezer-based trapping techniques [42]) have been discussed in [31] revealing how the mixture ${}^{23}$Na + ${}^{39}$K constitutes an almost ideal case which permits a smooth tuning of the interspecies and intraspecies scattering lengths and a considerable reduction in three-body losses. Furthermore, one can identify the ground-state phases of the system through the detection of the boson numbers at each well, for each component, effected by means of absorption-imaging techniques [43,44]. The experimental realization of these systems, in addition to the possibility of validating theoretical predictions on the separation mechanism, will enable the testing of the effectiveness of the BH-model picture in which potential wells, despite their small but finite extension, are approximated to point-like structures. The numerical study of the mixture in the three-well potential within the mean-field picture [31] has confirmed the validity of the BH-picture predictions.

In this paper, we derived the phase diagram of the four-well system and discussed its critical properties. We compared it with those of the two-well and three-well systems, and tried to highlight both possible common features that do not depend on the lattice size *L* and properties that instead reflect the influence of the degree of fragmentation in the transition from the full-mixing to the full-demixing regime (complete space separation). Our analysis suggests that, in addition to the full-mixing uniform solution, various other phases are expected to survive for a large *L*. This is the case, for example, of the SWM solutions (12), (15) and (18) (phases 2, 3 and 4 in Figure 1, Figure 2 and Figure 3, respectively) which correspond to state (27) for a generic *L*. Likewise, solutions (16) (phase 2 in Figure 2) can be associated, for arbitrary L=2s+1 and β≃1, to solution (23) with fully demixed components, except for a single well with residual mixing, while solution (24) generalizes, for arbitrary L=2s, solution (19) (phase 4 in Figure 3), showing *s* wells occupied by the majority component and *s* wells with residual mixing.

Solution (25), which describes full-demixing states for arbitrary *L* and sufficiently large α, represents the extension of solutions (20) and (21) (phases 6 and 7, respectively, in Figure 3), and has revealed that full demixing can be realized in L−2 different ways related to the asymmetry parameter β. This number, in turn, highlights how the partial-demixing region (featuring intermediate values of β∈[0,1] and α weak enough but such that α>1), must be equipped with a number of ground-state solutions sufficiently large in order to reproduce, for large α, the transitions to the extended set of full-demixing solutions. The architecture of this region is then expected to be really complex and to feature manifold ways to reach the full-demixing regime whose recognition is far from being trivial (see, e.g., the four-well phase diagrams for a preliminary example). This richness strongly resembles the scenario of ground-state configurations of quantum emulsions [15,45] and suggests the observation of quantum-granularity effects triggered by the interplay of strong effective interactions α, weak hopping amplitudes and fractional values of β [46]. These aspects will be further explored in a separate paper. 

## Data Availability

Not applicable.

## Abbreviations

The following abbreviations are used in this manuscript:

BH	Bose–Hubbard
CS	Coherent state
SWM	Single well mixing

## Appendix A. Four-Well Solutions of Equation (9)

The search for trial solutions to the four-well mixture equations, which exhibit different space distributions of the two components and different degrees of mixing and space separation, leading to consider the following configurations:

(A1) $$\left(1\right)\;x_{\ell }\neq 0,\;y_{\ell }\neq 0,$$

(A2) $$\left(2\right)\;x_{2}=y_{1}=0,\;x_{n},y_{k}\neq 0,\;\mathrm{for}\;n\neq 2,\;k\neq 1,$$

(A3) $$\left(3\right)\;x_{\ell }\neq 0,\;y_{1}\neq 0,\;\;\;y_{2},y_{3},y_{4}=0,$$

(A4) $$\left(4\right)\;x_{\ell }\neq 0,\;y_{2},y_{4}\neq 0,\;y_{1},y_{3}=0,$$

(A5) $$\left(5\right)\;y_{1}=0,\;y_{k}\neq 0\;\;\left(k\neq 1\right),\;x_{n}\neq 0,\;\forall n,$$

(A6) $$\left(6\right)\;y_{1},y_{3}\neq 0,\;\;x_{2},x_{4}\neq 0,\;y_{2},y_{4},x_{1},x_{3}=0,$$

(A7) $$\left(7\right)\;y_{1},x_{2},x_{3},x_{4}\neq 0,\;x_{1},y_{2},y_{3},y_{4}=0.$$

The final form of these trial solutions is determined by means of Equation (9). We sketch the solution of these seven cases.

*Case 1*. Solution (A1) was obtained by repeating the procedure applied to the two-well system. This shows that, despite the presence of parameters α, β, $\lambda_{a}$, and $\lambda_{b}$, one obtains $\rho_{\ell }=\rho_{k}$ and $\eta_{\ell }=\eta_{k}$ for any *ℓ* and *k*. The use of constraints (7) leads to the expected form:

(A8) $$\rho_{\ell }=\eta_{\ell }=1/4.$$

*Case 2*. More interestingly, solution (A2) entails the reduced set of equations $\lambda_{a}=\rho_{1}$, $\lambda_{b}=\eta_{2}$, and $\lambda_{a}=\rho_{k}+\alpha \beta \eta_{k}$, $\lambda_{b}=\eta_{k}+\alpha \rho_{k}/\beta$ with k=3,4, which allows one to show that $\rho_{3}=\rho_{4}$ and $\eta_{3}=\eta_{4}$ due to the symmetry in the index exchange 3↔4. Thanks to the constraints $1=\rho_{1}+2\rho_{3}$, $1=\eta_{2}+2\eta_{3}$, one finds:

(A9) $$\rho_{3}=\frac{3-\beta \alpha }{9-\alpha^{2}},\;\eta_{3}=\frac{3-\alpha /\beta }{9-\alpha^{2}}.$$

*Case 3*. Because of the form of solution (A3), Equation (9) reduce to $\lambda_{a}=\rho_{1}+\alpha \beta \eta_{1}$, $\lambda_{b}=\eta_{1}+\alpha \rho_{1}/\beta$ and $\lambda_{a}=$$\rho_{2}=\rho_{3}=\rho_{4}$. The resulting explicit form reads:

(A10) $$\rho_{1}=\frac{1-3\alpha \beta }{4},\;\rho_{k}=\frac{1+\alpha \beta }{4},\;\eta_{1}=1,\;\;\eta_{k}=0,$$

with k=2,3,4, where the two constraints $1=\rho_{1}+3\rho_{2}$, $1=\eta_{1}$ are used.

*Case 4*. Solution (A4) is characterized by fact that $\eta_{1}=\eta_{3}=0$ entails $\lambda_{a}=\rho_{1}=\rho_{3}$. Then, this case can be seen as a double dimer with residual mixing in one of the two wells. Equation (9) then reduces to a system of four equations:

$$\lambda_{a}=\rho_{2}+\alpha \beta \eta_{2},\;\lambda_{b}=\eta_{2}+\alpha \rho_{2}/\beta ,$$

$$\lambda_{a}=\rho_{4}+\alpha \beta \eta_{4},\;\lambda_{b}=\eta_{4}+\alpha \rho_{4}/\beta ,$$

which, in view of the symmetry in the index exchange 2↔4, entails $\rho_{2}=\rho_{4}$ and $\eta_{2}=\eta_{4}$. By exploiting the two constraints $1=\eta_{2}+\eta_{4}$ (giving $\eta_{2}=\eta_{4}=1/2$) and $1=2\rho_{1}+2\rho_{2}$, the resulting system formed by $\rho_{1}=\rho_{2}+\alpha \beta /2$ $\rho_{1}+\rho_{2}=1/2$ (with the chemical potential $\lambda_{b}=1/2+\alpha \rho_{2}/\beta$) provides the solution:

(A11) $$\rho_{1}=\frac{1+\alpha \beta }{4},\;\rho_{2}=\frac{1-\alpha \beta }{4},\;\eta_{1}=\eta_{2}=\frac{1}{2}.$$

*Case 5*. Solution (A5) features $\eta_{1}=0$ because of condition $y_{1}=0$. Apart from $\lambda_{a}=\rho_{1}$, the remaining six equations of system (9), once more due to symmetry in the index exchange k↔ℓ for any pair k,ℓ=2,3,4, reduce to the pair of equations:

$$\lambda_{a}=\rho_{2}+\alpha \beta \eta_{2},\;\lambda_{b}=\eta_{2}+\alpha \rho_{2}/\beta ,$$

with $\rho_{2}=\rho_{3}=\rho_{4}$ and $\eta_{2}=\eta_{3}=\eta_{4}$. The explicit solution is easily found to be:

(A12) $$\rho_{1}=\frac{1+\alpha \beta }{4},\;\rho_{2}=\frac{3-\alpha \beta }{12},\;\eta_{k}=\frac{1}{3},\;\;k=2,3,4.$$

*Case 6*. Solution (A6) can be reconducted to the fully demixed solutions of two twin dimers. System (9) reduces to $\lambda_{a}=\rho_{2}=\rho_{4}$ and $\lambda_{b}=\eta_{1}=\eta_{3}$ with $1=\eta_{1}+\eta_{3}$ and $1=\rho_{2}+\rho_{4}$. As in the derivation of solution (13), one has $\rho_{1}=\rho_{3}=\eta_{2}=\eta_{4}=0$ together with:

(A13) $$\rho_{2}=\rho_{4}=\frac{1}{2},\;\eta_{1}=\eta_{3}=\frac{1}{2}.$$

*Case 7*. Solution (A7) recasts system (9) into the form $\lambda_{a}=\rho_{k}$, k=2,3,4, $\lambda_{b}=\eta_{1}$ with the constraints $1=\eta_{1}$, $1=\rho_{2}+\rho_{3}+\rho_{4}$. Then:

(A14) $$\rho_{1}=0,\;\rho_{k}=\frac{1}{3},\;\eta_{1}=1,\;\eta_{k}=0,\;k=2,3,4.$$

## References

[1] Ao P., Chui S.T. (1998). Binary Bose-Einstein condensate mixtures in weakly and strongly segregated phases. Phys. Rev. A.

[2] Timmermans E. (1998). Phase Separation of Bose-Einstein Condensates. Phys. Rev. Lett..

[3] Jaksch D., Bruder C., Cirac J.I., Gardiner C.W., Zoller P. (1998). Cold Bosonic Atoms in Optical Lattices. Phys. Rev. Lett..

[4] Gersch H.A., Knollman G.C. (1963). Quantum Cell Model for Bosons. Phys. Rev..

[5] Fisher M.P.A., Weichman P.B., Grinstein G., Fisher D.S. (1989). Boson localization and the superfluid-insulator transition. Phys. Rev. B.

[6] Catani J., De Sarlo L., Barontini G., Minardi F., Inguscio M. (2008). Degenerate Bose-Bose mixture in a three-dimensional optical lattice. Phys. Rev. A.

[7] Gadway B., Pertot D., Reimann R., Schneble D. (2010). Superfluidity of Interacting Bosonic Mixtures in Optical Lattices. Phys. Rev. Lett..

[8] Soltan-Panahi P., Struck J., Hauke P., Bick A., Plenkers W., Meineke G., Becker C., Windpassinger P., Lewenstein M., Sengstock K. (2011). Multi-component quantum gases in spin-dependent hexagonal lattices. Nat. Phys..

[9] Mishra T., Pai R.V., Das B.P. (2007). Phase separation in a two-species Bose mixture. Phys. Rev. A.

[10] Lingua F., Guglielmino M., Penna V., Capogrosso Sansone B. (2015). Demixing effects in mixtures of two bosonic species. Phys. Rev. A.

[11] Kuklov A.B., Svistunov B.V. (2003). Counterflow Superfluidity of Two-Species Ultracold Atoms in a Commensurate Optical Lattice. Phys. Rev. Lett..

[12] Altman E., Hofstetter W., Demler E., Lukin M.D. (2003). Phase diagram of two-component bosons on an optical lattice. New J. Phys..

[13] Guglielmino M., Penna V., Capogrosso-Sansone B. (2011). Ising antiferromagnet with ultracold bosonic mixtures confined in a harmonic trap. Phys. Rev. A.

[14] Suthar K., Angom D. (2017). Characteristic temperature for the immiscible-miscible transition of binary condensates in optical lattices. Phys. Rev. A.

[15] Roscilde T., Cirac J.I. (2007). Quantum Emulsion: A Glassy Phase of Bosonic Mixtures in Optical Lattices. Phys. Rev. Lett..

[16] Buonsante P., Giampaolo S.M., Illuminati F., Penna V., Vezzani A. (2008). Mixtures of Strongly Interacting Bosons in Optical Lattices. Phys. Rev. Lett..

[17] Lingua F., Capogrosso-Sansone B., Minardi F., Penna V. (2017). Thermometry of bosonic mixtures in Optical Lattices via Demixing. Sci. Rep..

[18] Amico L., Osterloh A., Cataliotti F. (2005). Quantum Many Particle Systems in Ring-Shaped Optical Lattices. Phys. Rev. Lett..

[19] Aghamalyan D., Amico L., Kwek L.C. (2013). Effective dynamics of cold atoms flowing in two ring-shaped optical potentials with tunable tunneling. Phys. Rev. A.

[20] Amico L., Aghamalyan D., Auksztol F., Crepaz H., Dumke R., Kwek L.C. (2014). Superfluid qubit systems with ring shaped optical lattices. Sci. Rep..

[21] Albiez M., Gati R., Fölling J., Hunsmann S., Cristiani M., Oberthaler M.K. (2005). Direct Observation of Tunneling and Nonlinear Self-Trapping in a Single Bosonic Josephson Junction. Phys. Rev. Lett..

[22] Anker T., Albiez M., Gati R., Hunsmann S., Eiermann B., Trombettoni A., Oberthaler M.K. (2005). Nonlinear Self-Trapping of Matter Waves in Periodic Potentials. Phys. Rev. Lett..

[23] Lingua F., Mazzarella G., Penna V. (2016). Delocalization effects, entanglement entropy and spectral collapse of boson mixtures in a double well. J. Phys. B At. Mol. Opt. Phys..

[24] Lingua F., Penna V. (2017). Continuous-variable approach to the spectral properties and quantum states of the two-component Bose-Hubbard dimer. Phys. Rev. E.

[25] Pyzh M., Schmelcher P. (2020). Phase separation of a Bose-Bose mixture: Impact of the trap and particle-number imbalance. Phys. Rev. A.

[26] Lingua F., Richaud A., Penna V. (2018). Residual entropy and critical behavior of two interacting boson species in a double well. Entropy.

[27] Günay M. (2020). Binary mixture of Bose-Einstein condensates in a double-well potential: Berry phase and two-mode entanglement. Phys. Rev. A.

[28] Richaud A., Penna V. (2018). Phase separation can be stronger than chaos. New J. Phys..

[29] Syu W.C., Lee D.S., Lin C.Y. (2020). Regular and chaotic behavior of collective atomic motion in two-component Bose-Einstein condensates. Phys. Rev. A.

[30] Penna V., Richaud A. (2018). The phase separation mechanism of a binary mixture in a ring trimer. Sci. Rep..

[31] Richaud A., Zenesini A., Penna V. (2019). The mixing-demixing phase diagram of ultracold heteronuclear mixtures in a ring trimer. Sci. Rep..

[32] Burchianti A., D’Errico C., Prevedelli M., Salasnich L., Ancilotto F., Modugno M., Minardi F., Fort C. (2020). A dual-species Bose-Einstein condensate with attractive interspecies interactions. Condens. Matter.

[33] Richaud A., Penna V. (2019). Pathway toward the formation of supermixed states in ultracold boson mixtures loaded in ring lattices. Phys. Rev. A.

[34] Amico L., Penna V. (1998). Dynamical Mean Field Theory of the Bose-Hubbard Model. Phys. Rev. Lett..

[35] Buonsante P., Penna V. (2008). Some remarks on the coherent-state variational approach to nonlinear boson models. J. Phys. A Math. Theor..

[36] Oelkers N., Links J. (2007). Ground-state properties of the attractive one-dimensional Bose-Hubbard model. Phys. Rev. B.

[37] Buonsante P., Penna V., Vezzani A. (2011). Dynamical bifurcation as a semiclassical counterpart of a quantum phase transition. Phys. Rev. A.

[38] Pęcak D., Sowiński T. (2016). Few strongly interacting ultracold fermions in one-dimensional traps of different shapes. Phys. Rev. A.

[39] Penna V., Richaud A. (2017). Two-species boson mixture on a ring: A group-theoretic approach to the quantum dynamics of low-energy excitations. Phys. Rev. A.

[40] Brandani G.B., Schor M., MacPhee C.E., Grubmüller H., Zachariae U., Marenduzzo D. (2013). Quantifying disorder through conditional entropy: An application to fluid mixing. PLoS ONE.

[41] Camesasca M., Kaufman M., Manas-Zloczower I. (2006). Quantifying fluid mixing with the Shannon entropy. Macromol. Theory Simul..

[42] Kaufman A.M., Lester B.J., Regal C.A. (2012). Cooling a Single Atom in an Optical Tweezer to Its Quantum Ground State. Phys. Rev. X.

[43] Chomaz L., Corman L., Yefsah T., Desbuquois R., Dalibard J. (2012). Absorption imaging of a quasi-two-dimensional gas: A multiple scattering analysis. New J. Phys..

[44] Smith D.A., Aigner S., Hofferberth S., Gring M., Andersson M., Wildermuth S., Krüger P., Schneider S., Schumm T., Schmiedmayer J. (2011). Absorption imaging of ultracold atoms on atom chips. Opt. Express.

[45] Buonsante P., Giampaolo S., Illuminati F., Penna V., Vezzani A. (2009). Unconventional quantum phases in lattice bosonic mixtures. Eur. Phys. J. B.

[46] Richaud A., Penna V. (2020). Quantum-granularity effect in the formation of supermixed solitons in ring lattices. Condens. Matter.

